# Severe pneumonia in children with *influenza A*–*Staphylococcus aureus* coinfection: case report

**DOI:** 10.3389/fped.2026.1797919

**Published:** 2026-04-13

**Authors:** Pei Tao, Zhigang Wang, Yinghong Fan

**Affiliations:** Chengdu Women and Children's Central Hospital, School of Medicine, University of Electronic Science and Technology of China, Sichuan, China

**Keywords:** children, coinfection, *influenza A* virus, methicillin-resistant *Staphylococcus aureus*, necrotizing pneumonia

## Abstract

Severe community-acquired pneumonia caused by coinfection with *influenza A* virus and *Staphylococcus aureus* (*S. aureus*) is rare but may lead to rapid clinical deterioration and high mortality. We report two pediatric cases of severe pneumonia associated with *influenza A* virus-*S. aureus* coinfection. Both patients initially presented with nonspecific influenza-like symptoms, followed by rapid progression to acute respiratory failure. One case was characterized by necrotizing pneumonia complicated by pneumothorax and empyema, with *S. aureus* isolated from respiratory specimens and pleural fluid. The other case developed *S. aureus* bloodstream infection, progressing to septic shock and multiple organ dysfunction syndrome. Both children required prolonged invasive mechanical ventilation and multidisciplinary intensive care, including targeted antimicrobial therapy, organ support, and surgical intervention. Antimicrobial treatment consisted of vancomycin with adjunctive linezolid for 4–6 weeks. Despite severe disease, both patients achieved clinical recovery and were discharged, although residual pulmonary abnormalities were observed on follow-up. These cases highlight the heterogeneous clinical presentations of influenza-associated *S. aureus* coinfection in children. Early recognition, timely optimization of antimicrobial therapy, and comprehensive supportive management are essential to improve outcomes in this potentially life-threatening condition.

## Introduction

Pneumonia is one of the most common respiratory diseases in children and remains the leading infectious cause of morbidity and mortality among children under five years of age worldwide. In 2019, approximately 740,000 children younger than five years died from pneumonia globally (1), and data from 2022 indicate that the global incidence of pediatric pneumonia exceeded 14% (2). The majority of pediatric pneumonia cases are classified as community-acquired pneumonia, with viruses, bacteria, and atypical pathogens representing the principal etiologic agents. Notably, mixed infections are frequently observed in children with pneumonia, with an overall prevalence of approximately 20%–30% (3, 4), and the likelihood of coinfection increases with decreasing age. Viral–bacterial coinfections have been reported across all pediatric age groups (5).

Surveillance data from the Chinese Center for Disease Control and Prevention indicate that influenza virus is the most commonly detected pathogen in acute respiratory tract infections among school-aged children (5–17 years) in China, with a detection rate of 18.75%, and it is also among the three leading causes of community-acquired pneumonia in children. Among children aged 0–5 years, the detection rate of influenza virus is 11.11%, while in those aged 6–17 years it is 11.69%. *Staphylococcus aureus* (*S. aureus*) is a well-recognized pathogen capable of causing severe, often necrotizing pneumonia in children and is associated with substantial mortality. In recent years, coinfection with *influenza A* virus and *S. aureus*, particularly community-associated methicillin-resistant *S. aureus* (CA-MRSA), has been reported with increasing frequency during influenza epidemics (6) and is associated with rapid clinical deterioration and poor outcomes in pediatric patients (7, 8). Against this background, we describe two children with influenza A complicated by CA-MRSA pneumonia, bacteremia, septic shock, acute respiratory distress syndrome, and multiple organ dysfunction, both of whom showed favorable responses to timely and aggressive treatment.

## Case 1

A 7-year-old boy was admitted to the intensive care unit with a two-day history of fever, followed by one day of lethargy, fatigue, and progressively worsening dyspnea, accompanied by cough and reduced appetite. His past medical, birth, and developmental history were unremarkable. He had not received seasonal influenza vaccination prior to illness onset. Shortly after admission, his respiratory condition worsened rapidly, requiring endotracheal intubation and mechanical ventilation.

**Figure 1:**
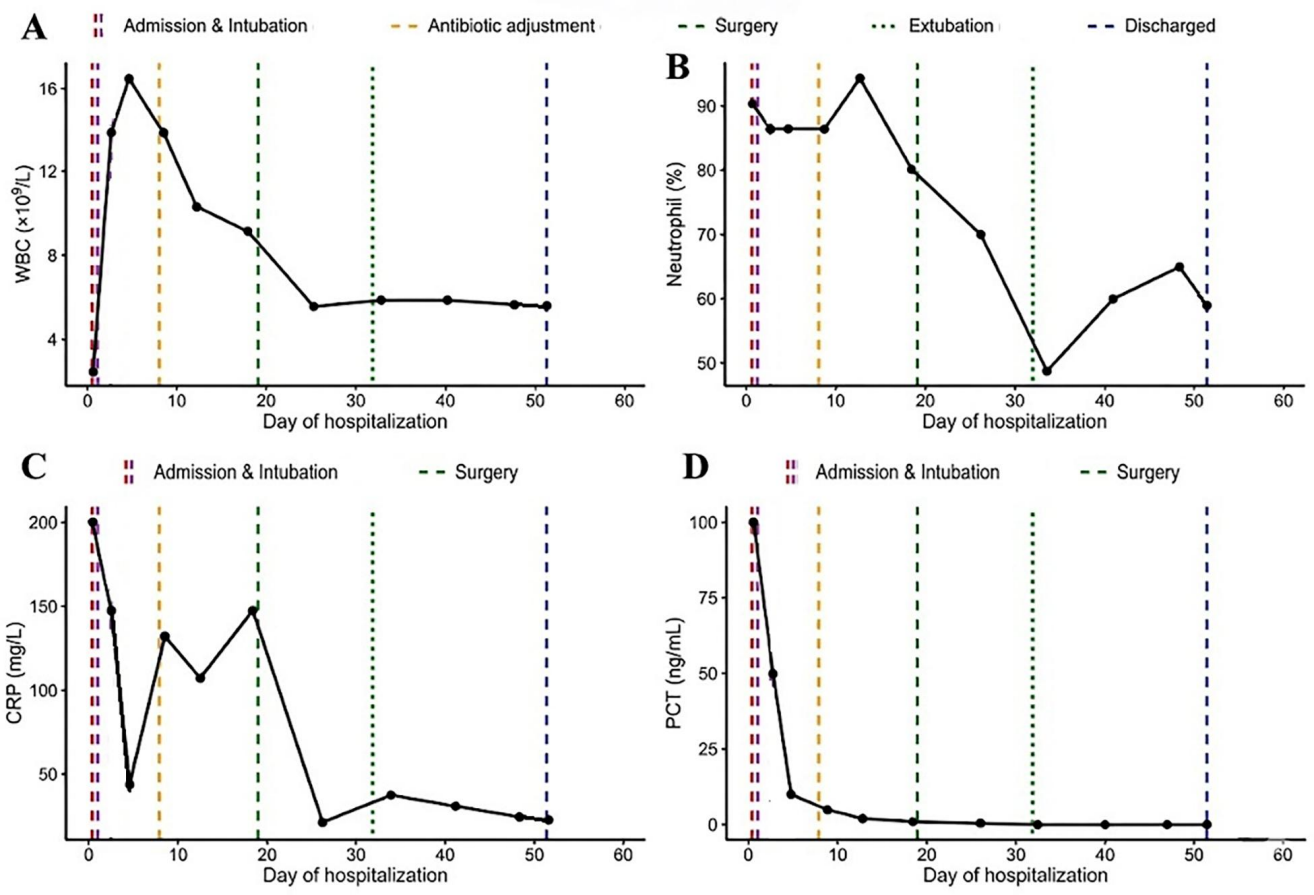
Temporal profiles of inflammatory biomarkers in a pediatric patient with severe pneumonia. **(A)** White blood cell count (WBC); **(B)** neutrophil percentage (*N*%); **(C)** C-reactive protein (CRP), and **(D)** procalcitonin (PCT) levels. The *x*-axis represents days since hospital admission. Colored lines indicate key clinical events: red dashed line for admission, purple dashed line for endotracheal intubation, orange dashed line for antimicrobial adjustment, green dashed line for surgical intervention, green dotted line for extubation, and blue dashed line for discharge.

Laboratory tests showed fluctuating white blood cell counts (Figure 1A), persistently elevated neutrophil percentages (Figure 1B), and markedly increased C-reactive protein and procalcitonin levels (Figures 1C, D). Analysis of inflammatory cytokines and lymphocyte subsets revealed marked abnormalities during the acute phase. Interleukin-6 (IL-6) and interleukin-10 (IL-10) were markedly elevated at 8,077.15 pg/mL and 875.43 pg/mL, respectively. Interleukin-17A (IL-17A) was also increased (23.18 pg/mL), whereas tumor necrosis factor-α (TNF-α) remained within the reference range (5.46 pg/mL). Lymphocyte subset analysis demonstrated reduced natural killer (NK) cells (5.3%) and markedly elevated B cells (50.6%). During the convalescent phase, cytokine levels and lymphocyte distributions gradually returned toward normal ranges (Table 1).

**Table 1:** Changes in inflammatory cytokines and lymphocyte subsets between acute and convalescent phases.

Parameter	Unit	Acute phase	Convalescent phase	Reference range	Fold change^†^	Change (%)
IL-6	pg/mL	8,077.15	490.59	0–7	1,154× ↑	−93.9
IL-10	pg/mL	875.43	18.23	0–9.1	96× ↑	−97.9
TNF-α	pg/mL	5.46	0.61	0–8.1	0.11× ↓	−88.8
IL-17A	pg/mL	23.18	3.88	0–15	1.5× ↑	−83.3
NK cells	%	5.30	7.90	7–40	0.7× ↓	+49.1
B cells	%	50.60	33.40	5–18	2.8× ↑	−34.0

† Fold change was calculated as the ratio of acute-phase values to the upper limit of the reference range. Percentage change represents the relative difference between acute and convalescent phases. ↑ indicates an increase, and ↓ indicates a decrease.

**Figure 2:**
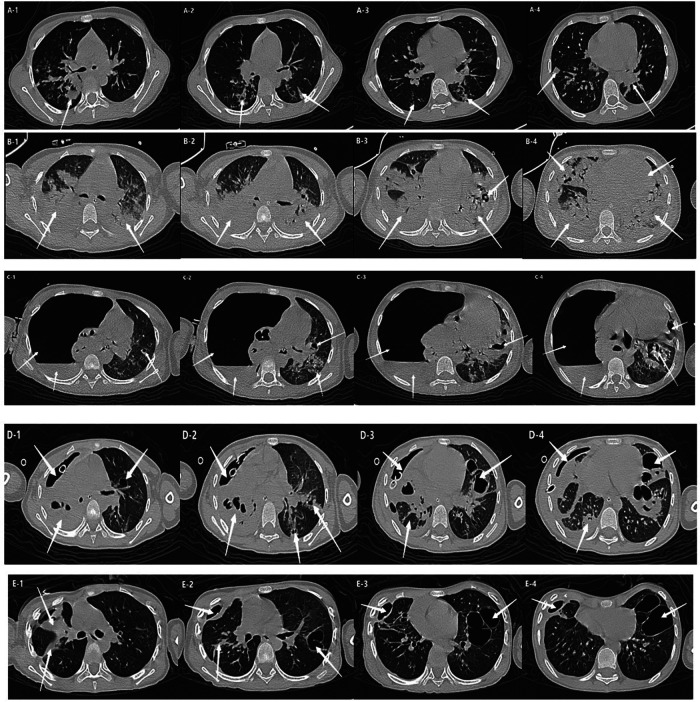
Chest CT: 1) **(A1-A4)**: Multiple bilateral patchy, flocculent, and flaky density opacities. 2) **(B1-B4)** Diffuse multifocal patchy and consolidative opacities in both lungs with air bronchograms. Multiple scattered cavitary lesions of variable size. Small bilateral pleural effusions. 3) **(C1-C4)** Right hemithorax shows extensive gas density with minimal fluid, causing right lung compression and atelectasis. Collapsed lung contains multiple air-fluid cavities and interstitial emphysema-like changes. Left lung demonstrates patchy consolidations with variably-sized cavities along bronchovascular bundles. 4) **(D1-D4)**: Trachea and mediastinum slightly shifted to the right. Right lung exhibits extensive pneumothorax and minimal hydrothorax with compression and atelectasis, multiple air-fluid cavities, and interstitial emphysema-like patterns. Right lower lobe shows bronchial gas trapping. Left lung shows patchy consolidations with variably-sized cavities, some containing air-fluid levels. Extensive subcutaneous emphysema in right neck, chest wall, axilla, and abdominal wall. 5) **(E1-E4)**: Right pleural cavity shows linear area without lung markings and adjacent pleural thickening. Patchy and band-like opacities in right middle and upper lobes with air bronchograms and local bronchial dilation. Scattered flocculent hazy opacities in both lower lobes.

Chest CT showed progressive bilateral pulmonary involvement that worsened during the acute course. Following treatment, pulmonary lesions gradually resolved, with residual giant pulmonary cysts observed (Figure 2).

**Figure 3:**
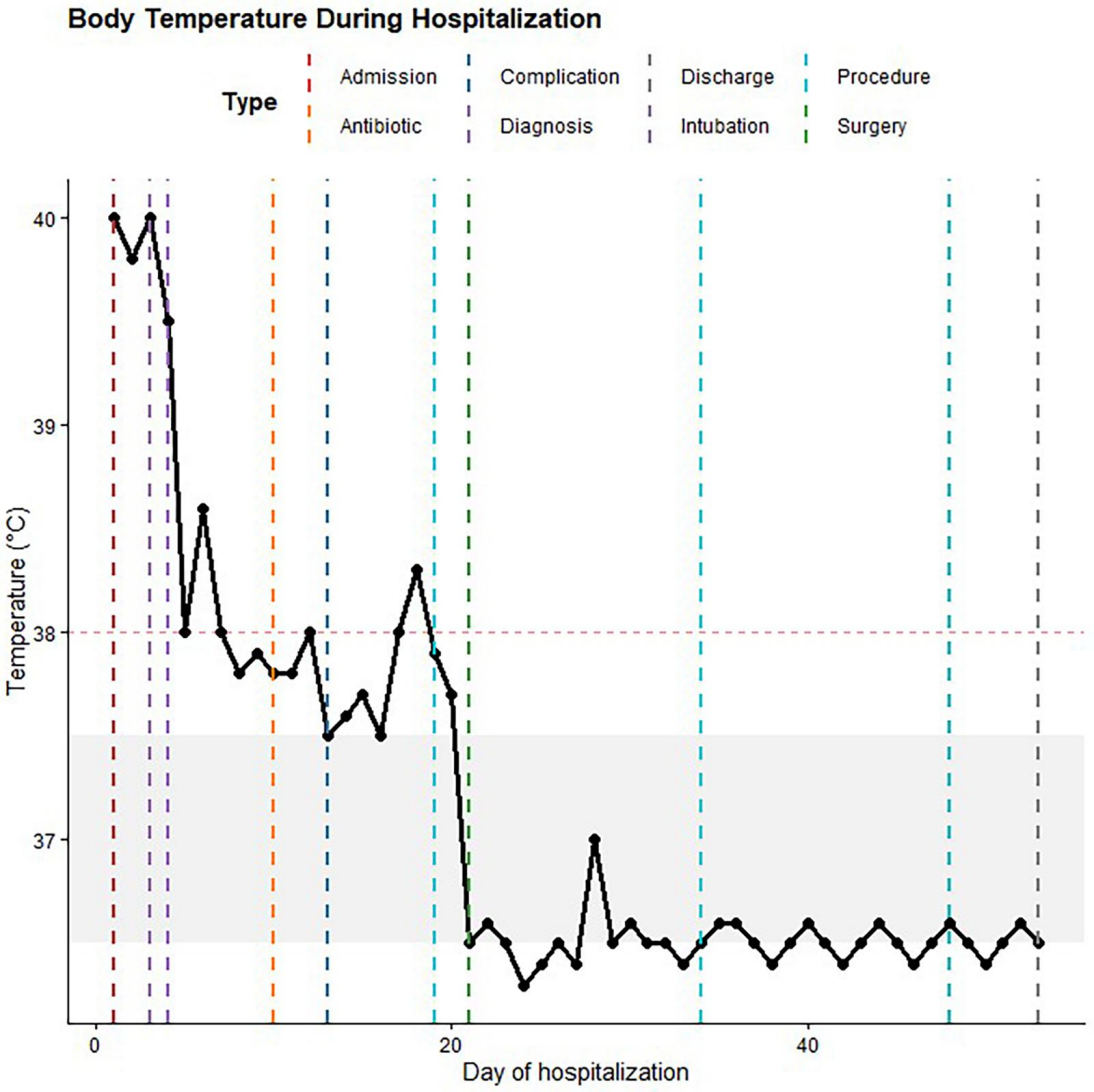
Temporal trend of body temperature during hospitalization.

Empirical treatment was initiated with cefoperazone–sulbactam, inhaled budesonide, and comprehensive supportive care, including organ support and hemodynamic stabilization. Antiviral therapy with peramivir was started after influenza A nucleic acid testing returned positive. Despite these interventions, the patient remained febrile (Figure 3). Although inflammatory markers showed a partial decline, the patient continued to exhibit persistent fever and clinical instability, and follow-up chest CT demonstrated radiologic progression, including expansion of pulmonary consolidation, enlargement of necrotic cavities, and development of empyema. These findings met our criteria for persistent infection and clinical treatment failure, defined as ongoing fever, sustained inflammatory response, and lack of radiological improvement despite appropriate antimicrobial therapy. Given the extensive pulmonary involvement and the need for improved pulmonary tissue penetration in severe necrotizing pneumonia, linezolid was therefore added as adjunctive anti-MRSA therapy. The patient subsequently underwent surgical evacuation with closed thoracic drainage, after which fever resolved and organ function progressively recovered. Mechanical ventilation was successfully discontinued after approximately 31 days of invasive ventilatory support. During this period, ventilator settings were adjusted dynamically according to clinical condition and blood gas analysis, with a gradual transition from controlled ventilation to assisted modes prior to successful weaning. The prolonged duration of mechanical ventilation was attributed to severe necrotizing pneumonia, persistent infection, and pleural complications requiring surgical intervention. Tracheostomy was not performed.

Etiological testing confirmed influenza A (H1N1) infection. Methicillin-resistant *Staphylococcus aureus* (MRSA) was repeatedly isolated from sputum and pleural pus cultures during hospitalization. Antimicrobial susceptibility testing demonstrated that the isolate was susceptible to vancomycin and linezolid, with a vancomycin minimum inhibitory concentration (MIC) ranging from ≤0.5 to 1 μg/mL and a linezolid MIC of 2 μg/mL, while resistance to clindamycin and erythromycin was observed. Molecular typing further identified the strain as community-associated MRSA (CA-MRSA) ST59-SCCmec IV-t437, which is consistent with previously reported pediatric isolates in China (9, 10).

The patient was diagnosed with severe community-acquired pneumonia caused by influenza A (H1N1) and MRSA coinfection, complicated by acute respiratory failure and empyema. Following combined antiviral therapy, targeted antimicrobial treatment, respiratory support, and surgical intervention, the patient achieved clinical stabilization, successful weaning from mechanical ventilation, and gradual radiological improvement, although residual pulmonary cystic changes persisted.

The key clinical events are summarized in Table 2.

**Table 2:** Chronological timeline of clinical events and management in case 1.

Illness day	Hospital day	Date	Clinical events
Day 1	–	∼2023-03-04	Onset of fever, fatigue, and dyspnea.
Day 3	Day 1	2023-03-06	Admitted to PICU with severe pneumonia and respiratory failure; endotracheal intubation and invasive mechanical ventilation initiated.
Day 4	Day 2	2023-03-07	*Influenza A* virus infection confirmed
Day 10	Day 8	2023-03-13	Methicillin-resistant *Staphylococcus aureus* (MRSA) isolated from respiratory culture
Day 13	Day 11	2023-03-16	Acute clinical deterioration with right pneumothorax; closed thoracic drainage performed.
Day 19	Day 17	2023-03-22	Chest CT revealed necrotizing pneumonia with empyema
Day 21	Day 19	2023-03-24	Thoracoscopic pleural adhesion release and empyema debridement performed
Day 34	Day 32	2023-04-06	Successfully extubated following respiratory improvement.
Day 48	Day 46	2023-04-20	Chest drainage tube removed
Day 53	Day 51	2023-04-25	Discharged after clinical recovery.

## Case 2

**Figure 4:**
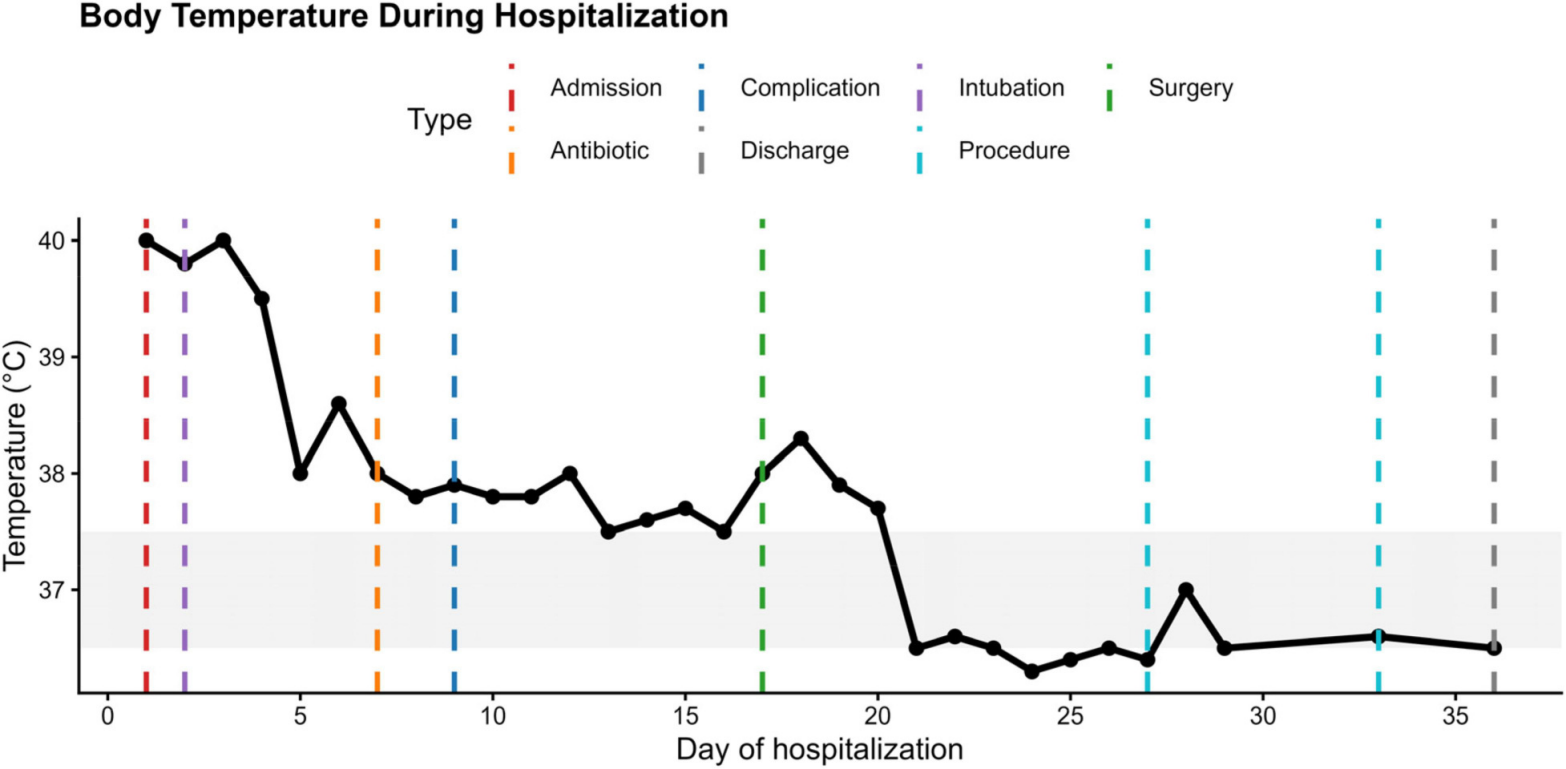
Temporal trend of body temperature during hospitalization.

An 8-year-old girl was admitted with a one-day history of high fever and cough, accompanied by diarrhea for half a day, wheezing, headache, and abdominal pain. Her past medical, birth, and developmental history were unremarkable. She had not received seasonal influenza vaccination prior to illness onset. Shortly after admission, she developed persistent fever (Figure 4) and progressive respiratory failure, requiring immediate endotracheal intubation and invasive mechanical ventilation.

**Figure 5:**
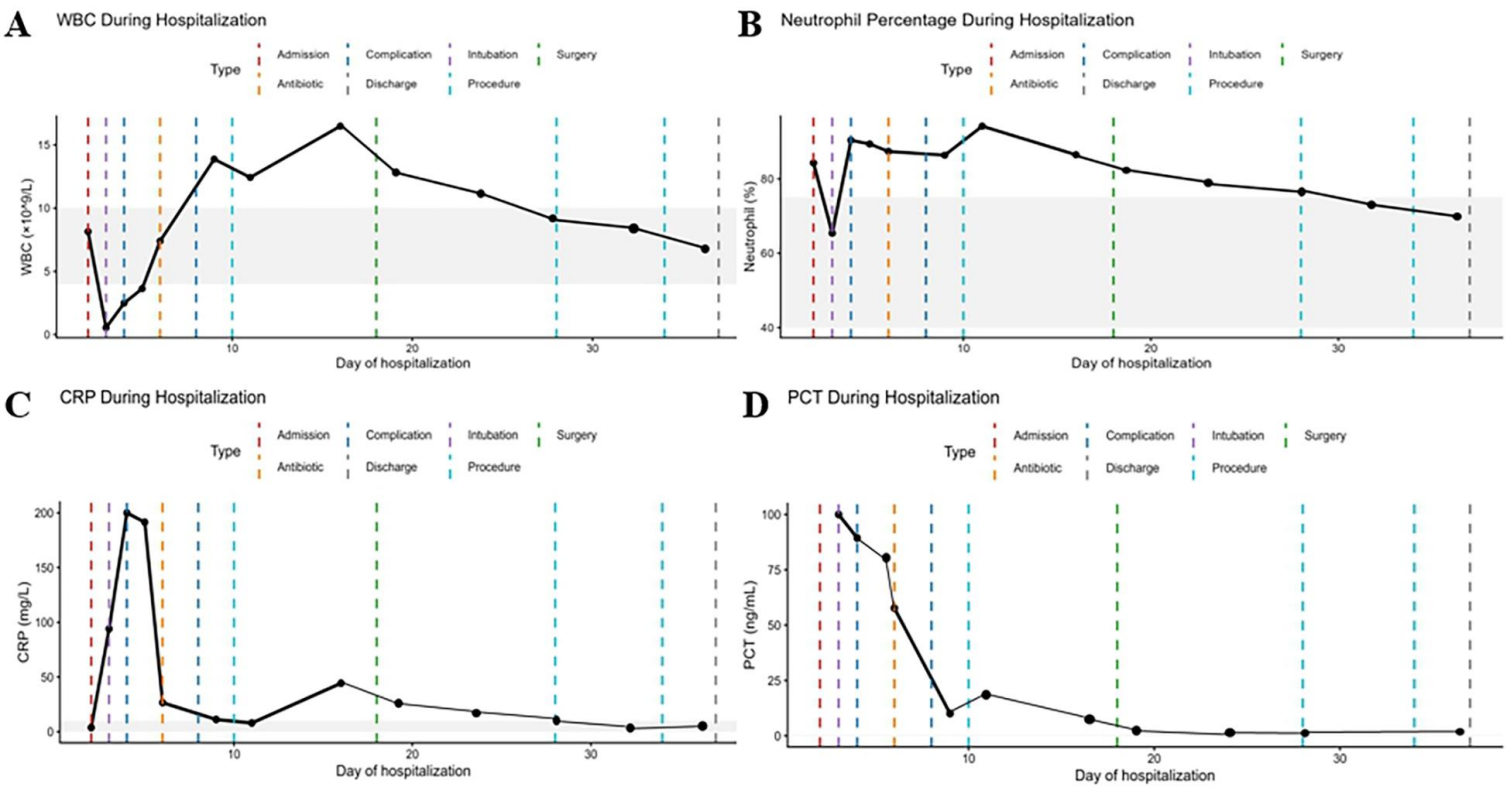
Temporal profiles of inflammatory biomarkers during hospitalization. **(A)** White blood cell count (WBC); **(B)** neutrophil percentage (NEU); **(C)** C-reactive protein (CRP), and **(D)** procalcitonin (PCT) were dynamically monitored throughout the hospital course. The shaded gray areas indicate the normal reference ranges for each parameter. Colored dashed vertical lines represent key clinical events, including hospital admission, initiation of antibiotic therapy, development of complications, endotracheal intubation, surgical intervention, procedures, and discharge.

Laboratory investigations initially revealed leukopenia followed by leukocytosis (Figure 5A), with persistently elevated neutrophil percentages (Figure 5B) and markedly increased C-reactive protein and procalcitonin levels (Figures 5C,D). Analysis of inflammatory cytokines and lymphocyte subsets indicated pronounced immune dysregulation during the acute phase. Interleukin-6 (IL-6) and interleukin-10 (IL-10) were markedly elevated, reaching 8,077.15 pg/mL and 3,393.02 pg/mL, respectively. Interleukin-17A (IL-17A) showed a mild increase (8.03 pg/mL), whereas tumor necrosis factor-α (TNF-α) remained within the reference range (3.34 pg/mL). Lymphocyte subset analysis demonstrated reduced natural killer (NK) cells (4.3%) and a marked expansion of B cells (55.8%). During the recovery phase, cytokine concentrations and lymphocyte distributions gradually approached normal ranges (Table 3).

**Table 3:** Changes in inflammatory cytokines and lymphocyte subsets between acute and convalescent phases.

Parameter	Unit	Acute phase	Convalescent phase	Reference range	Fold change†	Change (%)
IL-6	pg/mL	8,077.15	14.23	0–7	1,154×↑	−99.8
IL-10	pg/mL	3,393.02	3.19	0–9.1	373×↑	−99.9
TNF-α	pg/mL	4.96	0.89	0–8.1	0.18× ↓	−82.1
IL-17A	pg/mL	8.03	2.24	0–15	0.28× ↓	−72.1
NK cells	%	4.3	7.9	7–40	1.8×↑	+83.7
B cells	%	55.8	37.5	5–18	3.1×↑	−32.8

† Fold change was calculated as the ratio of acute-phase values to the upper limit of the reference range. Percentage change represents the relative difference between acute and convalescent phases. ↑ indicates an increase, and ↓ indicates a decrease.

**Figure 6:**
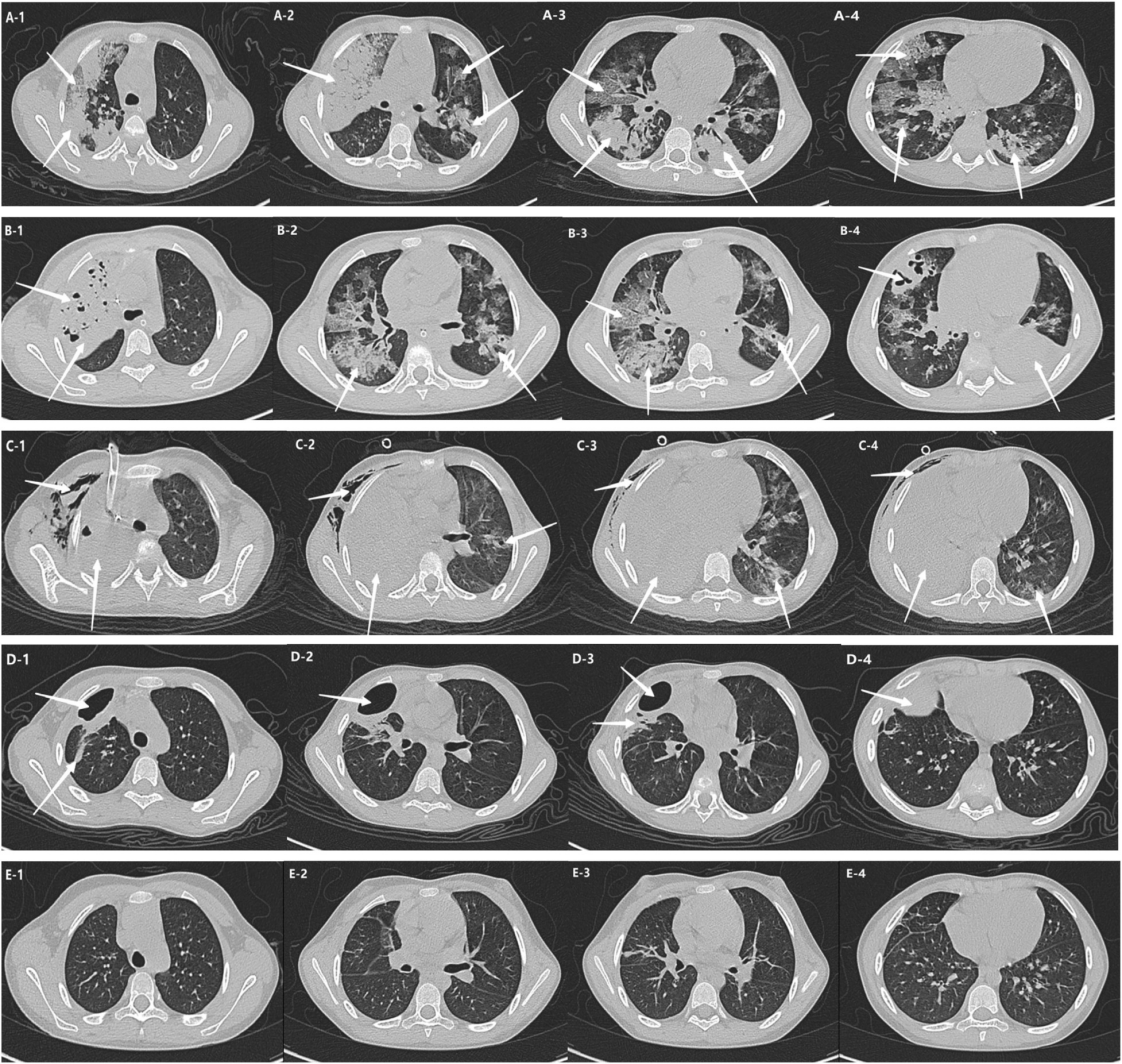
1) **(A1-A4)**: Multiple large consolidations and ground-glass opacities in both lungs, with air bronchograms and scattered pneumatoceles. 2) **(B1-B4)**: Extensive consolidation with volume loss in the right upper lobe and left lower lobe, more pronounced in the left lower lobe. Multiple patchy and nodular ground-glass opacities are seen along the peribronchovascular bundles. Pneumatoceles are noted within involved areas, predominantly in the right upper lobe. 3) **(C1-C4)**: E Right lung shows extensive consolidation with irregular cystic and patchy low-density opacities and scattered small cystic lucencies. Left lung demonstrates patchy ground-glass opacities and band-like increased densities, with small cystic lucencies in the left upper lobe apical-posterior segment. Subcutaneous air collections are present in the chest wall. 4) **(D1-D4)**: A linear lucency devoid of lung markings is seen in the right pleural cavity with adjacent pleural thickening. Patchy and band-like opacities are present in the right middle and upper lobes with air bronchograms. Local bronchial dilation and traction are noted. Scattered patchy, hazy opacities are observed in both lower lobes. 5) **(E1-E4)**: Scattered fibrous stripes in the right lung, consistent with residual chronic inflammatory changes.

Chest CT demonstrated rapidly progressive bilateral pulmonary involvement during the acute course. Initial imaging showed extensive consolidation and ground-glass opacities with air bronchograms, accompanied by multiple pneumatoceles. As the disease progressed, the areas of consolidation enlarged and were associated with cystic or patchy low-density lesions, scattered pneumatoceles, and subcutaneous emphysema of the chest wall. After treatment, pulmonary abnormalities gradually improved, although residual necrosis, cavitation, and fibrotic streaks persisted, consistent with chronic inflammatory changes (Figure 6).

Empirical antimicrobial therapy with cefotaxime and vancomycin was initiated on admission. After confirmation of influenza virus infection, oseltamivir was started according to standard antiviral recommendations (2 mg/kg twice daily for 5 days). The patient subsequently underwent thoracoscopic empyema debridement with closed thoracic drainage. Following these interventions, her clinical condition gradually stabilized and invasive mechanical ventilation was successfully discontinued.

Invasive mechanical ventilation was discontinued after approximately 16 days of ventilatory support. Ventilator settings were adjusted dynamically according to clinical condition and blood gas analysis, with gradual transition from controlled ventilation to assisted modes prior to successful weaning. The prolonged duration of ventilation was attributed to severe lung injury, systemic inflammation, and pleural complications. Tracheostomy was not performed.

Etiological evaluation confirmed influenza A (H3N2) infection by rapid antigen testing of a nasopharyngeal swab. Blood and pleural fluid cultures yielded *Staphylococcus aureus*. Antimicrobial susceptibility testing identified methicillin-sensitive *Staphylococcus aureus* (MSSA), which was susceptible to oxacillin, vancomycin, linezolid, and clindamycin.

The patient was diagnosed with severe community-acquired pneumonia caused by influenza A (H3N2) and *Staphylococcus aureus* coinfection, complicated by acute respiratory failure and empyema. With timely respiratory support, targeted antimicrobial therapy, and surgical management, the patient achieved clinical stabilization and gradual radiological improvement, although residual structural lung abnormalities were observed during follow-up.

The key clinical events are summarized in Table 4.

**Table 4:** Chronological timeline of clinical events and management in case 2.

Illness day	Hospital day	Date	Clinical events
Day 1	–	2023-03-10	Fever, cough, dyspnea, abdominal pain, and diarrhea began.
Day 2	Day 1	2023-03-11	Admitted to the PICU with severe influenza A infection, severe pneumonia, and acute respiratory failure.
Day 3	Day 2	2023-03-12	Endotracheal intubation and invasive mechanical ventilation initiated; septic shock, ARDS, and multiple organ dysfunction developed. First plasma exchange performed.
Day 4	Day 3	2023-03-13	Myocardial dysfunction identified on echocardiography; second plasma exchange performed.
Day 6	Day 5	2023-03-15	Third plasma exchange performed; hemodynamic status gradually stabilized.
Day 8	Day 7	2023-03-17	*Staphylococcus aureus* isolated from blood culture; antimicrobial therapy adjusted.
Day 10	Day 9	2023-03-19	Right pneumothorax developed; emergency thoracic drainage was performed.
Day 18	Day 17	2023-03-27	Thoracoscopic pleural adhesion release/debridement and closed thoracic drainage performed for persistent pleural disease and pneumothorax.
Day 18	Day 17	2023-03-27	Extubated after respiratory improvement and switched to oxygen therapy.
Day 28	Day 27	2023-04-06	Bronchoscopy with bronchoalveolar lavage performed for airway clearance; pulmonary infection further improved.
Day 34	Day 33	2023-04-11	Chest drainage tube removed.
Day 37	Day 36	2023-04-14	Discharged after clinical recovery.

In both cases, leukocyte counts initially decreased and subsequently increased, with persistently elevated neutrophil proportions. Inflammatory markers, including C-reactive protein (CRP) and procalcitonin (PCT), rose markedly during the acute phase. Chest imaging demonstrated rapid progression within 3–5 days, with extensive consolidation and partial lung collapse. Clinically, both patients presented with prolonged fever and severe disease requiring intensive care.Despite these shared features, the clinical course and severity differed between the two cases. To better illustrate these similarities and differences, the key clinical characteristics are summarized in Table 5.

**Table 5:** Case 1 vs. Case 2.

Feature	Case 1	Case 2
Pathogen	MRSA (respiratory)	MSSA (blood)
Shock	No	Yes
CRRT	No	Yes
Surgical intervention	Yes	Yes
Mechanical ventilation	31 days	16 days
Complications	Pneumothorax, empyema	Shock, MODS

## Discussion

Coinfection with influenza virus (IV) and *S. aureus*, particularly methicillin-resistant *S. aureus* (MRSA), is recognized as a severe clinical condition associated with substantially increased morbidity and mortality compared with influenza infection alone (11–13). Previous studies have reported that secondary bacterial infections contributed to a large proportion of influenza-related deaths during past pandemics, with *S. aureus* being one of the most frequently identified pathogens (11, 12). In pediatric populations, an increasing number of severe or fatal influenza cases complicated by *S. aureus* infection, including MRSA, have also been reported (12, 14).

Influenza-associated *S. aureus* coinfection is often characterized by rapid clinical deterioration and severe pulmonary complications, including necrotizing pneumonia, respiratory failure, and septic shock (14–17). Mortality rates in such cases remain considerable, particularly when necrotizing pneumonia develops. Previous reports have described mortality rates ranging from approximately 20% to more than 50% in patients with influenza-associated MRSA pneumonia (15, 16). The clinical features observed in our patients were largely consistent with those reported in the literature, including abrupt respiratory deterioration, extensive pulmonary necrosis with cavitary changes on imaging, and markedly elevated inflammatory markers.

The pathogenesis of influenza-associated *S. aureus* infection involves complex interactions between viral-induced host immune dysfunction and bacterial virulence factors. Influenza infection disrupts epithelial barrier integrity and impairs innate immune defenses, thereby facilitating bacterial colonization and invasion. Experimental studies have demonstrated that influenza impairs neutrophil function, enhances bacterial adherence through upregulation of host adhesion molecules such as fibronectin and integrins, and activates inflammatory pathways (18–20). In addition, *S. aureus* virulence factors, including Panton–Valentine leukocidin (PVL), contribute to neutrophil destruction and severe lung injury, promoting the development of necrotizing pneumonia (21, 22). However, PVL status was not assessed in this study, which represents a limitation, as PVL is considered an important virulence factor in necrotizing pneumonia.

In China, the epidemiology of MRSA among pediatric patients has also evolved in recent years. Community-associated MRSA (CA-MRSA) has become increasingly prevalent, with ST59-SCCmec IV-t437 emerging as the dominant clone (23). Previous molecular epidemiological studies have shown that this lineage is widely distributed among pediatric infections in China. In the present study, the MRSA isolate identified in one patient belonged to the ST59-SCCmec IV-t437 clone, which is consistent with these earlier reports. The isolate remained susceptible to vancomycin and linezolid, which likely contributed to the favorable clinical response observed in this patient. Importantly, the identification of this high-virulence MRSA clone further strengthens the pathogen-specific interpretation of disease severity and supports its potential role in driving necrotizing pneumonia in this case.

Children with influenza complicated by *S. aureus* infection are at increased risk of acute respiratory distress syndrome (ARDS), severe lung injury, and systemic inflammatory responses (24). In both of our cases, rapid progression of pulmonary inflammation and early respiratory failure were observed. Chest imaging demonstrated extensive consolidation, necrosis, and cavitary changes, while laboratory investigations revealed markedly elevated inflammatory markers, including white blood cell counts, neutrophil percentages, C-reactive protein, and procalcitonin levels.

Experimental studies suggest that influenza infection predisposes the host to secondary bacterial invasion through several immunological mechanisms. These include impairment of neutrophil phagocytic activity, increased bacterial adherence mediated by host adhesion molecules, and activation of inflammatory pathways such as TGF-*β* signaling (18, 20). Coinfection models have also demonstrated significant elevations of proinflammatory cytokines, including IL-6 and TNF-α, reflecting intense innate immune activation (25). Consistent with these findings, our patients exhibited pronounced inflammatory responses during the acute phase, characterized by markedly elevated cytokine levels and alterations in immune cell subsets. The substantial elevation of IL-6 and IL-10 likely reflects systemic immune activation and dysregulation during severe viral–bacterial coinfection.

Influenza-associated bacterial coinfection may also affect adaptive immune responses. Previous studies have shown that influenza infection can suppress Th17-mediated host defense and reduce recruitment of natural killer (NK) cells, contributing to immune dysregulation during secondary bacterial infection (26). In line with these observations, our patients demonstrated early abnormalities in cellular immune profiles, including reduced NK cell proportions and altered B-cell distribution.

The two cases in our study illustrate the clinical spectrum of influenza-associated *S. aureus* coinfection in children. One patient developed severe necrotizing pneumonia caused by CA-MRSA belonging to the ST59-SCCmec IV-t437 clone, whereas the other patient had invasive infection caused by methicillin-sensitive *S. aureus* (MSSA). Despite differences in antimicrobial resistance profiles, both patients experienced rapid disease progression and severe pulmonary injury.

Our experience also suggests that favorable outcomes can still be achieved in severe cases when early intensive care support, targeted antimicrobial therapy, and timely surgical management of complications are provided. Despite the severity of illness and the presence of multiple complications, both patients survived following comprehensive multidisciplinary management.

These cases highlight the rapid progression and severe pulmonary injury that may occur in influenza-associated *S. aureus* coinfection. Early recognition of this condition, careful monitoring of inflammatory and immune parameters, and prompt initiation of appropriate antimicrobial and supportive therapy are likely critical for improving clinical outcomes. Nevertheless, residual pulmonary sequelae were observed during follow-up, indicating that severe coinfections may still result in long-term respiratory morbidity.

## Limitations

This study has several limitations. First, the sample size was small and derived from a single center, which may limit generalizability. Second, the retrospective design introduces potential selection and information bias. Third, not all relevant virulence factors (e.g., PVL status) and host-related factors (e.g., vaccination history) were systematically assessed. Finally, causal relationships between pathogens and clinical outcomes cannot be definitively established.

## Conclusion

Severe pneumonia caused by influenza and *Staphylococcus aureus* coinfection in children can progress rapidly and result in significant pulmonary injury. Clinicians should maintain a high index of suspicion for bacterial coinfection during influenza seasons, particularly in patients with rapid clinical deterioration or necrotizing pulmonary lesions. Early pathogen identification and appropriate antimicrobial therapy remain essential for improving clinical outcomes.

## Data Availability

The original contributions presented in the study are included in the article/supplementary material, further inquiries can be directed to the corresponding author.
